# ifDEEPre: large protein language-based deep learning enables interpretable and fast predictions of enzyme commission numbers

**DOI:** 10.1093/bib/bbae225

**Published:** 2024-06-28

**Authors:** Qingxiong Tan, Jin Xiao, Jiayang Chen, Yixuan Wang, Zeliang Zhang, Tiancheng Zhao, Yu Li

**Affiliations:** Department of Computer Science and Engineering, The Chinese University of Hong Kong, Hong Kong SAR, China; Department of Computer Science, Hong Kong Baptist University, Hong Kong SAR, China; Department of Computer Science and Engineering, The Chinese University of Hong Kong, Hong Kong SAR, China; Department of Computer Science and Engineering, The Chinese University of Hong Kong, Hong Kong SAR, China; Department of Computer Science, University of Rochester, Rochester, New York State, USA; School of Computer Science and Technology, Huazhong University of Science and Technology, Wuhan, China; School of Software, Shandong University, Jinan, China; Department of Computer Science and Engineering, The Chinese University of Hong Kong, Hong Kong SAR, China; The CUHK Shenzhen Research Institute, Nanshan, Shenzhen, China

**Keywords:** enzyme prediction, large language model, evolutionary inference, protein motif detection, interpretability analysis, self-guided attentive learning

## Abstract

Accurate understanding of the biological functions of enzymes is vital for various tasks in both pathologies and industrial biotechnology. However, the existing methods are usually not fast enough and lack explanations on the prediction results, which severely limits their real-world applications. Following our previous work, *DEEPre*, we propose a new interpretable and fast version (*ifDEEPre*) by designing novel self-guided attention and incorporating biological knowledge learned via large protein language models to accurately predict the commission numbers of enzymes and confirm their functions. Novel self-guided attention is designed to optimize the unique contributions of representations, automatically detecting key protein motifs to provide meaningful interpretations. Representations learned from raw protein sequences are strictly screened to improve the running speed of the framework, 50 times faster than DEEPre while requiring 12.89 times smaller storage space. Large language modules are incorporated to learn physical properties from hundreds of millions of proteins, extending biological knowledge of the whole network. Extensive experiments indicate that ifDEEPre outperforms all the current methods, achieving more than 14.22% larger F1-score on the NEW dataset. Furthermore, the trained ifDEEPre models accurately capture multi-level protein biological patterns and infer evolutionary trends of enzymes by taking only raw sequences without label information. Meanwhile, ifDEEPre predicts the evolutionary relationships between different yeast sub-species, which are highly consistent with the ground truth. Case studies indicate that ifDEEPre can detect key amino acid motifs, which have important implications for designing novel enzymes. A web server running ifDEEPre is available at https://proj.cse.cuhk.edu.hk/aihlab/ifdeepre/ to provide convenient services to the public. Meanwhile, ifDEEPre is freely available on GitHub at https://github.com/ml4bio/ifDEEPre/.

## INTRODUCTION

Enzymes are essential catalysts in every species, known to increase chemical reactions under biological conditions and play key roles in many biological processes, e.g. energy conversion, nutrition and metabolism [1, 2]. Annotating the functions of enzymes is vital because it can promote their real-world applications, e.g. the diagnosis of enzyme-related diseases, and the design of new effective enzymes [3]. Any malfunction of enzymes, no matter underproduction or overproduction, may lead to a series of severe diseases and damages. The deficiency of Phenylalanine Hydroxylase often causes Phenylketonuria, which destabilizes protein structures and could lead to Attention-Deficit/Hyperactivity disorder problems if no treatment is given [4]. Conversely, high-level Transaminase causes the failure in maintaining normal cell functions of the liver for breaking down substances and removing toxins from bodies, illustrating potential liver damages [5]. Accurate understanding of enzyme biological functions is crucial for identifying triggers of these diseases and developing enzyme-based effective treatments [6].

Traditional methods often conduct wet laboratory-based experiments to confirm enzyme functions, e.g. enzymatic assays [7], which require large domains of expertise and are very time-consuming. Thus, these methods are often used for small-sized protein databases. Because the abundance and near-exponential growth of new enzymes, e.g. structures of enzymes registered in Protein Data Bank are over 186 000, laboratory-based methods are becoming less practical. The most well-known way of annotating enzyme functions is the enzyme commission (EC) system, which is a tree structure consisting of four digits that most specifically describe the main classes, the chemical bond, the chemical reactions and the substrates of enzymes [8]. The first digit to the fourth digit of EC, respectively, indicates whether a protein is an enzyme, and provides its sub-class, sub-sub-class and sub-sub-sub-class if it is an enzyme. By precisely predicting EC numbers, the functions of enzymes can be annotated accordingly, associating the protein with specific chemical reactions [9].

Computational methods have been used for EC number prediction for decades. Based on the belief that the functions of proteins are often determined by their structures, several methods first predict protein structures and then obtain the EC numbers, e.g. I-TASSER [10]. Specifically, after the structure prediction stage, the protein databases that contain the entire EC numbers, confirmed via experiments, are scanned. Thus, each protein sequence is assigned with the EC numbers of the sequences in the databases that have the most similar structure. However, the structure predictions of these methods are usually slow and time-consuming, which thus are not suitable for large protein databases. Even worse, there are errors at both the structure prediction and the EC number confirmation stages, which would be accumulated and thus significantly limit the final prediction performances.

Secondly, some models are built by considering similarities between proteins. Their common assumption is that sequences of enzymes with highly similar biological functions should meanwhile have large similarities. They first select the enzyme sequences with a given EC number and then achieve the goal of dividing sequences in a target protein database into different sub-groups by considering their similarity with the known enzyme [11]. However, these models require significant homologies in current databases for each input protein sequence that needs to be estimated. As a result, they may fail to perform accurate function predictions for protein sequences without such homologies in the known databases, which however is a very common issue for newly discovered enzyme proteins.

Machine learning models [12–15] are widely built to first extract features from raw sequences and then predict EC numbers. Typical methods include Ezypred [16], Svm-prot [17], ABLE [8] and ProPythia [18]. However, they often need to use manually crafted features as inputs, requiring the devotion of a large volume of time and energy of experts, which may fail to meet the needs of handling large quantities of proteins [17]. Although recent methods can extract features from raw sequences in an automatic way, most of them rely heavily on usages of PSI-BLAST from BLAST+ to extract Position-Specific Scoring Matrix (PSSM) features through multiple iterations, e.g. DEEPre [9]. Thus, they still need long time to obtain PSSM features, failing to meet the needs of handling a large quantity of proteins in the post-genomic era.

Another two issues severely limit further applications of these methods. First, the existing methods are not able to provide meaningful *interpretations* on their results, which makes them hard to be applied in reality to promote public health. This is because protein sequences are typically complex and difficult to analyze. Thus, when models perform predictions without providing any interpretation, it would be difficult for bio-engineering scientists to have a good understanding of the results and accordingly improve their designs. The absence of interpretations meanwhile reduces the reliability of obtained results, further limiting their applications. Secondly, current enzyme prediction models often fail to fully utilize important *biological knowledge* of proteins contained in super quantities of *unlabeled protein sequences* to improve their results. There are already hundreds of millions of proteins in current databases and an increasing number of new sequences are being discovered each day, which contain vital information on the physical properties of proteins spanning evolutionary diversity and are crucial for enzyme predictions. However, such crucial biological knowledge is often ignored by the existing methods, which severely impacts their prediction performances.

To tackle the above challenges, following our previous work *DEEPre*, we propose a new *i*nterpretable and *f*ast version, *ifDEEPre*, for accurate EC number predictions by designing novel self-guided attention and incorporating vital protein biological knowledge learned from hundreds of millions of proteins. To effectively optimize the contribution weights of different representations according to the unique properties of tasks, we design new self-guided attention to dynamically adjust the weights of all the elements. This mechanism automatically reinforces the roles of key elements and detects important motifs to provide meaningful interpretations, assisting biological scientists in designing more effective strategies. Meanwhile, to meet the needs of modeling millions of proteins in the post-genomic era, representations learned from raw sequences are strictly screened to improve the speed of the framework, e.g. excluding manually crafted features [17] and PSSM [9], which take long to extract. Signal channel of each representation is constructed under a residual learning structure via short-cut connection units, further facilitating the information propagation and stabilizing the network training. Furthermore, to fully utilize the advantages of the big data era, we introduce a large language module [19] into this framework to capture vital biological knowledge from hundreds of millions of proteins. This design incorporates rich types of biological knowledge into ifDEEPre, e.g. function information, mutational effects and folding knowledge, not only boosting performances but also promoting downstream applications. Extensive experimental results and analysis suggest the superiority of the proposed ifDEEPre method in various tasks, including enzyme function predictions, model interpretability analysis, multi-level protein knowledge learning and evolutionary relationship inferences.

## MATERIALS AND METHODS

**Figure 1:**
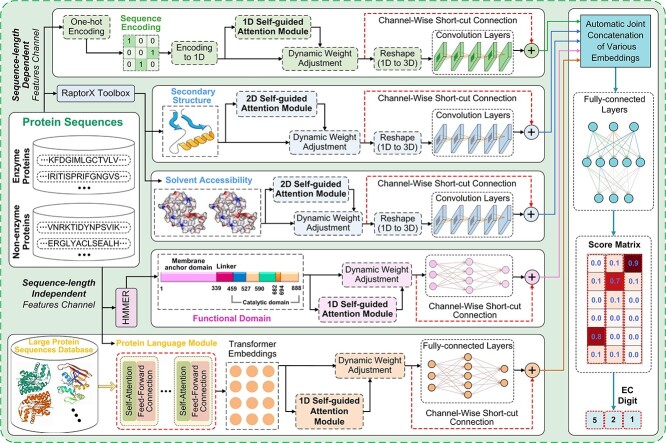
Architecture of ifDEEPre for accurate enzyme predictions. Both sequence-length-dependent and -independent embeddings are automatically learned to capture rich knowledge from raw sequences for enzyme predictions. Self-guided attention and short-cut connections are simultaneously introduced into convolution layers for sequence-length-dependent features, dynamically optimizing contributions of various elements and stabilizing training of the whole network. Meanwhile, fully connected layers with self-guided attention and short-cut connections are designed for sequence-length-independent features to adjust the weights of all the representations, which effectively promotes the distillation of global biological knowledge learned from millions of proteins to improve accuracy. Learnable fusion classifiers finally concatenate all the refined embeddings to obtain final results.

The architecture of the proposed method is presented in Figure 1. Sequence-length-dependent and -independent embeddings are simultaneously learned to capture rich protein knowledge. Self-guided attentive residual learning structures are designed to dynamically optimize contributions of different representations while stabilizing the training of the framework. Embeddings extracted from various aspects are then injected into a learnable fusion layer to obtain final enzyme prediction results. We first introduce the data collection and pre-processing process.

### Data collection and pre-processing

In this study, we conduct experiments on several benchmark datasets, i.e. NEW dataset from [9], KNN dataset from [16] and COFACTOR dataset from [20]. NEW dataset is constructed from SWISS-PROT database by Li *et al*. [9], which excludes sequences with incomplete or more than one set of EC numbers and limits sequence similarities at 40% to remove redundancy bias, resulting in 22 168 non-enzymes and 22 168 enzymes with 3343 Oxidoreductases, 8517 Transferases, 5917 Hydrolases, 1532 Lyases, 1193 Isomerases and 1666 Ligases. For KNN dataset, conditions in [16] are used to process data, obtaining 9850 non-enzymes and 9832 enzymes that consist of 1618 Oxidoreductases, 3450 Transferases, 2791 Hydrolases, 679 Lyases, 518 Isomerases and 776 Ligases. For both datasets, we randomly split 80%, 10% and 10% of sequences into training data, validation data and testing data.

Besides, a third-party independent and non-overlapping protein dataset is used to evaluate the generalization capacity of the proposed ifDEEPre model. Therefore, the non-homologous dataset, i.e. the COFACTOR dataset from [20], is utilized for the cross-dataset validation experiments, which meets the following three requirements: (a) the similarities of protein sequences in this dataset are smaller than 30%; (b) this dataset does not contain any self-BLAST hit and has no homologous enzymes; (c) the overlap sequences between the training data (the NEW dataset) and test dataset are removed from COFACTOR. Finally, 281 enzymes are obtained.

### Large protein language module

We learn two types of representations, i.e. sequence-length-dependent (sequence encoding, secondary structure and solvent accessibility) and independent features (functional domain and transformer features). Among them, sequence encoding, secondary structure, solvent accessibility and functional domain are obtained via techniques in [9], which merely consider biological properties in small-scaled training samples, limiting the width and depth of knowledge. Although millions of proteins are available and provide a large volume of valuable information on protein properties, learning biological knowledge from such a giant number of sequences is challenging. This is because existing models are often built upon supervised learning, requiring label information for model training, which however is not available for most protein sequences.

To tackle this issue, we incorporate a large language model, ESM-1b [19], as a key module for accurate enzyme predictions by learning valuable biological properties from millions of proteins, obtaining transformer features to effectively extend knowledge of the neural network. This module is built upon the unsupervised learning mechanism, which outperforms traditional models and has become a powerful network architecture for learning informative representations. It consists of several self-attention and feed-forward blocks, which consider context across inputs. A large protein database UniParc with 250 million sequences and 86 billion amino acids across life is used to optimize this language module. The masked language modeling (MLM) objective is built to optimize parameters, which improves the capacity of the network in predicting token amino acids by capturing global sequential information from corrupted protein sequences: 


(1) \begin{align*}& \mathcal{L}_{{MLM}} = \mathbb{E}_{x\sim X} \mathbb{E}_{M} \sum_{i \in M} \log p (x_{i} | x_{/M}),\end{align*}

where $X$ denotes the whole dataset; $x$ is a sequence in $X$; $M$ represents the indices of the token mask; $x_{i}$ and $x_{/M}$ denote the true amino acid and the masked sequence, which are the prediction targets and the inputs of this module.

When training, errors between truly masked amino acids and predicted ones are fed back to this module, guiding it to adjust parameters by precisely capturing sequential patterns of proteins. The learned transformer embeddings reflect multi-scale protein biological properties, e.g. secondary structure, evolutionary information, function information and mutational effects, effectively extending biological knowledge of the whole network and enhancing its generalization ability. This language module runs fast, needing much less time than manually crafted [17] and PSSM features [9], so this module can effectively improve prediction accuracy while avoiding increasing the computational burden of the whole framework.

### Self-guided attention mechanism

Since these representations are learned from diverse aspects, e.g. sequence encoding conveys sequential information of proteins while secondary structure indicates local folding patterns of sequences, they may possess different degrees of importance for enzyme function predictions. We hence design novel self-guided attention and introduce this learning mechanism into each channel to dynamically optimize the contributions of different features and motifs while providing interpretations on the prediction results. To tackle the shape diversity issue, 1D self-guided attention is designed for sequence encoding, functional domain and transformer features, while the 2D version of attention is adopted for secondary structure and solvent accessibility. We denote the 1D representation as ${\boldsymbol{R}}^{{1D, 1t}}=[R^{{1D, 1t}}_{1}, R^{{1D, 1t}}_{2}, \cdots , R^{{1D, 1t}}_{{L}}]$, where ${1t}$ is ${seqd}$, ${fund}$ or ${traf}$, respectively, representing sequence encoding, functional domain or transformer features; ${L}$ is length of the representation. Different from vanilla attention, which learns weights from random status and may fail to achieve the global optimum, the proposed self-guided attention mechanism learns more accurate weights by fully considering the unique properties of data in each representation. 1D self-guided attention weights $\boldsymbol{\alpha }^{{1D, 1t}}$ are thus learned from ${\boldsymbol{R}}^{{1D,1t}}$ via 


(2) \begin{align*}& \boldsymbol{\alpha}^{{1D, 1t}} = \sigma({\boldsymbol{W}}^{{1D, 1t}}*{\boldsymbol{R}}^{{1D, 1t}} + {\boldsymbol{b}}^{{1D, 1t}}),\end{align*}

where ${\boldsymbol{W}}^{{1D, 1t}} \in{\boldsymbol{R}}^{L \times L}$ denotes a trainable weight matrix; ${\boldsymbol{b}}^{{1D, 1t}} \in{\boldsymbol{R}}^{L}$ is a trainable bias vector.

The learned attention weights $\boldsymbol{\alpha }^{{1D, 1t}}$ are then applied to adjust the contribution score of every element in the feature representation ${\boldsymbol{R}}^{{1D,1t}}$ via the expression 


(3) \begin{align*}& {\boldsymbol{R}}^{{1D, 1t}}_{att} = \boldsymbol{\alpha}^{{1D, 1t}} \cdot{\boldsymbol{R}}^{{1D, 1t}},\end{align*}

where $\cdot $ represents the elementwise multiplication operation.

Attention weights $\boldsymbol{\alpha }^{{1D, 1t}}$ are globally optimized according to unique properties of data in the representation ${\boldsymbol{R}}^{{1D, 1t}}$ and the back-propagation information of current prediction errors, which provides larger space to search for better optimal solutions. Meanwhile, $\boldsymbol{\alpha }^{{1D, 1t}}$ dynamically optimizes the contributions of elements in ${\boldsymbol{R}}^{{1D, 1t}}$ to improve weights of key fragments. We denote a 2D representation as ${\boldsymbol{R}}^{{2D, 2t}}=[R^{{2D, 2t}}_{1, 1}, R^{{2D, 2t}}_{1, 2}, \cdots , R^{{2D, 2t}}_{{1, L}}; \cdots ; R^{{2D, 2t}}_{m, 1}, R^{{2D, 2t}}_{m, 2},\cdots , R^{{2D, 2t}}_{{m, L}}]$, where ${2t}$ is *ss* or *acc*, respectively, representing secondary structure or solvent accessibility; ${L}$ is length of the 2D representation; $m$ is the dimension of the vertical axis. 2D self-guided attention weights $\boldsymbol{\alpha }^{{2D, 2t}}$ are learned from ${\boldsymbol{R}}^{{2D, 2t}}$ and meanwhile utilized to adjust contribution scores of different elements in this representation: 


(4) \begin{align*} & \boldsymbol{\alpha}^{{2D, 2t}} = \sigma({\boldsymbol{W}}^{{2D, 2t}}*{\boldsymbol{R}}^{{2D, 2t}} + {\boldsymbol{b}}^{{2D, 2t}}), \end{align*}

(5) \begin{align*} & {\boldsymbol{R}}^{{2D, 2t}}_{att} = \boldsymbol{\alpha}^{{2D, 2t}} \cdot{\boldsymbol{R}}^{{2D, 2t}}, \end{align*}

where ${\boldsymbol{W}}^{{2D, 2t}} \in{\boldsymbol{R}}^{m \times m}$ and ${\boldsymbol{b}}^{{1D, 1t}} \in{\boldsymbol{R}}^{m}$, respectively, denote trainable weight matrix and bias vector.

Note that the self-guided attention modules introduced into different signal channels are trained jointly to achieve global optimal. This is because they can effectively learn accurate and reliable attention weights from the unique characteristics of data properties while closely exchanging their learned key information through the back-propagation mechanism, dynamically adjusting their learning status according to the status of other channels and the whole neural network to achieve better results. More importantly, the learned attention weights can illustrate the contribution score of every element in the sequences of all the representations, providing meaningful interpretations on the prediction results. This is vital for promoting the real-world applications of the proposed method because such capacity helps researchers and doctors to have a good understanding of the input protein sequences and identify key fragments from a whole long protein sequence, assisting them in improving their designs.

### Channel-wise learning in residual structure

Sequence-length-dependent features and sequence-length-independent features exhibit diverse properties, i.e. the former type changes along lengths of input sequences while lengths of the latter one are fixed, so we adopt different machine learning components to handle them, learning dimension-unified features that can be conveniently modeled by any type of standard classifier. To tackle these diversities, CNN components are designed to learn convolutional information from the sequence-length-dependent features, e.g. sequence encoding, secondary structure and solvent accessibility, while FNN components are simultaneously introduced to analyze sequence-length-independent features, e.g. functional domain and transformer features. Thus, multiple CNN layers are introduced into each signal channel of sequence-length-dependent features to jointly model them.

Among all the sequence-length-dependent features, the shapes of secondary structure and solvent accessibility are 2D, while those of sequence encoding are 1D. For 2D representations, their features after being processed by the self-guided attention mechanism are obtained via Eq.(5), ${\boldsymbol{R}}^{{2D, 2t}}_{att}$, where ${2t}$ could be *ss* or *acc*, which, respectively, denotes secondary structure or solvent accessibility. For sequence encoding, the features after being processed by the self-guided attention mechanism are calculated via Eq.(3), ${\boldsymbol{R}}^{{1D, 1t}}_{att}$, where ${1t}$ is *seqd*, which represents sequence encoding. Each type of sequence-length-dependent feature is then reshaped into 3D to meet the shape requirement of convolutional units and is further modeled by CNN components to learn dimension-unified features ${\boldsymbol{R}}^{{3t}}_{unified}$: 


(6) \begin{align*} & {\boldsymbol{R}}^{{3t}}_{att, re}= \begin{cases} {\boldsymbol{R}}^{{seqd}}_{att, re} = {RS}^{{1D} \rightarrow{3D}}({\boldsymbol{R}}^{{1D, 1t}}_{att}), \text{if {${1t}$} = {$seqd$}},\\{\boldsymbol{R}}^{{ss}}_{att, re} = {RS}^{{2D} \rightarrow{3D}}({\boldsymbol{R}}^{{2D, 2t}}_{att}), \text{if {${2t}$} = {$ss$}},\\{\boldsymbol{R}}^{{acc}}_{att, re} = {RS}^{{2D} \rightarrow{3D}}({\boldsymbol{R}}^{{2D, 2t}}_{att}), \text{if {$\textit{2t}$} = {$acc$}}, \end{cases} \end{align*}

(7) \begin{align*} & {\boldsymbol{R}}^{{3t}}_{{unified}} = {CNN}({\boldsymbol{R}}^{{3t}}_{att, re}), \end{align*}

where ${3t}$ is $seqd$, $ss$ or $acc$; ${Reshape}^{{1D} \rightarrow{3D}}$ and ${Reshape}^{{2D} \rightarrow{3D}}$ are operations of reshaping feature from 1D to 3D and from 2D to 3D; ${CNN}$ represents the CNN components.

Because all the sequence-length-independent features are 1D representations, i.e. functional domain and transformer features, FNN components are thus introduced to each signal channel of these features to analyze them jointly. The sequence-length-independent features after being processed by the self-guided attention module is obtained via Eq.(3) ${\boldsymbol{R}}^{{1D, 1t}}_{att}$, where ${1t}$ could be *fund* or *traf*, which, respectively, represents functional domain features or transformer features. Since the shapes of these two sequence-length-independent features already meet the requirement of FNN units, we directly utilize FNN components to model them without any adjustments on shapes: 


(8) \begin{align*}& {\boldsymbol{R}}^{{1t}}_{unified} = {FNN}({\boldsymbol{R}}^{{1D, 1t}}_{att}),\end{align*}

where ${1t}$ is *fund* or *traf*; *FNN* is the FNN component.

However, these channels are constructed upon the deep learning theories containing many layers, which may potentially impair the propagation of information along the whole signal channel and limit the prediction accuracy of the final results. To tackle this issue, we perform channel-wise learning in residual structure to effectively facilitate the propagation of important information and stabilize the training of the deep neural network by introducing a short-cut connection structure into each channel, further improving enzyme predictions. Due to the diversity between shapes of extracted dimension-unified features and that of original representations, traditional short-cut connections cannot be applied. We hence adopt a one-layer fully connected network to transform the shape of the short-cut branch to the same with that of unified features. In CNN and FNN modules, several batch normalization operations are adopted to limit the value of learned features in the range [0, 1]. To keep the value unity of features in the short-cut branches and the main channel, we connect a batch normalization layer to the one-layer fully connected short-cut network. The reshaping operations are conducted for feature representations in all the CNN channels. Specifically, 2D representations, i.e. secondary structure and solvent accessibility, are flattened to 1D, while this operation is not performed for 1D features, i.e. sequence encoding. The short-cut features are thus learned and then combined with reshaped features via 


(9) \begin{align*} & {\boldsymbol{R}}^{{flatten, 3t}}_{att}= \begin{cases} {\boldsymbol{R}}^{{flatten, seqd}}_{att} = {\boldsymbol{R}}^{{1D, 1t}}_{att}, \text{if {${1t}$} = {$seqd$}}, \\{\boldsymbol{R}}^{{flatten, ss}}_{att} = {flatten}({\boldsymbol{R}}^{{2D, 2t}}_{att}), \text{if {${2t}$} = {$ss$}}, \\{\boldsymbol{R}}^{\textit{flatten, acc}}_{att} = \textit{flatten}({\boldsymbol{R}}^{\textit{2D, 2t}}_{att}), \text{if {$\textit{2t}$} = {$acc$}}, \end{cases} \end{align*}

(10) \begin{align*} & {\boldsymbol{R}}^{\textit{short, 3t}} = \textit{BN}(\textit{relu}({\boldsymbol{W}}^{\textit{short, 3t}}*{\boldsymbol{R}}^{\textit{flatten, 3t}}_{att} + {\boldsymbol{b}}^{\textit{short, 3t}})), \end{align*}

(11) \begin{align*} & {\boldsymbol{R}}^{\textit{combined, 3t}} = {\boldsymbol{R}}^{\textit{3t}}_{\textit{unified}} + {\boldsymbol{R}}^{\textit{short, 3t}}, \end{align*}

where $\textit{3t}$ is *seqd*, *ss* or *acc*; *flatten* denotes flatten operation; for *ss* and *acc*, ${\boldsymbol{W}}^{\textit{short, 3t}} \in{\boldsymbol{R}}^{L_{2*m} \times d_{u}}$ is a trainable weight matrix, where $L_{2}$ denotes the length of the representation and $m$ equals to 3; for *seqd*, ${\boldsymbol{W}}^{\textit{short, 3t}} \in{\boldsymbol{R}}^{L_{1} \times d_{u}}$ is a trainable weight matrix, where $L_{1}$ is the length of the representation; $d_{u}$ represents the dimension of the unified features, including both ${\boldsymbol{R}}^{\textit{1t}}_{\textit{unified}}$ and ${\boldsymbol{R}}^{\textit{3t}}_{\textit{unified}}$; ${\boldsymbol{b}}^{\textit{short, 3t}} \in{\boldsymbol{R}}^{d_{u}}$ is a trainable bias vector; *batchNorm* represents the batch normalization operation; *relu* denotes the Relu activation function.

Similar structures except for flatten operations are also designed and adopted for the FNN channels. This is because the functional domain or transformer input features and their FNN outputs have the same dimension, i.e. 1D representations: 


(12) \begin{align*} & {\boldsymbol{R}}^{\textit{short, 1t}} = \textit{BN}(relu({\boldsymbol{W}}^{\textit{short, 1t}}*{\boldsymbol{R}}^{\textit{1d, 1t}}_{att} + {\boldsymbol{b}}^{\textit{short, 1t}})), \end{align*}

(13) \begin{align*} & {\boldsymbol{R}}^{\textit{combined, 1t}} = {\boldsymbol{R}}^{\textit{1t}}_{unified} + {\boldsymbol{R}}^{\textit{short, 1t}}, \end{align*}

where $\textit{1t}$ is *fund* or *traf*; ${\boldsymbol{W}}^{\textit{short, 1t}} \in{\boldsymbol{R}}^{L_{1} \times d_{u}}$ is a trainable weight matrix; $L_{1}$ is feature length; $d_{u}$ is dimension of unified features; ${\boldsymbol{b}}^{\textit{short, 1t}} \in{\boldsymbol{R}}^{d_{u}}$ is a trainable bias vector; *batchNorm* denotes batch normalization; *relu* is Relu activation function.

### Embeddings and outputs

Embeddings learned from different channels are projected to the same dimensionality, i.e. dimension $d_{u}$ of unified features is set as 256, and can be conveniently concatenated. Specifically, signals in the three-dimensional channel outputted by the convolution layers are flattened to one-dimensional, which are then injected into several fully connected layers and obtain a one-dimensional feature with 256 elements. As for the one-dimensional channel, these representations are further refined by several fully connected layers and finally obtain a one-dimensional representation with 256 elements. We also tried other numbers of elements for the unified features, e.g. 64, 128,and 512, but their results are very similar.

These embeddings learned from various aspects are finally concatenated as an embedding $\textit{y}_{\textit{emb}}$ of dimension 1024 through another trainable neural network, which is expected to contain a large volume of valuable biological knowledge and can be applied to perform various downstream tasks. The embedding $\textit{y}_{\textit{emb}}$ is finally injected into a fully connected classifier to obtain the EC number prediction result $\textit{y}_{\textit{pred}}$: 


(14) \begin{align*} & \begin{aligned} \textit{y}_{\textit{emb}} = \textit{BN}(\textit{relu}({\boldsymbol{W}}^{\textit{seqd}}*{\boldsymbol{R}}^{\textit{emb, seqd}} + {\boldsymbol{W}}^{\textit{ss}}*{\boldsymbol{R}}^{\textit{emb, ss}} + \\{\boldsymbol{W}}^{\textit{acc}}*{\boldsymbol{R}}^{\textit{emb, acc}}+{\boldsymbol{W}}^{\textit{fund}}*{\boldsymbol{R}}^{\textit{emb, fund}} + \\{\boldsymbol{W}}^{\textit{traf}}*{\boldsymbol{R}}^{\textit{emb, traf}} + {\boldsymbol{b}}^{\textit{emb}})), \end{aligned} \end{align*}

(15) \begin{align*} & \textit{y}_{\textit{pred}} = \textit{softmax}({\boldsymbol{W}}^{\textit{pred}}*\textit{y}_{\textit{emb}} + {\boldsymbol{b}}^{\textit{pred}}), \end{align*}

where ${\boldsymbol{W}}^{\textit{seqd}} \in{\boldsymbol{R}}^{d_{u} \times 1024}$, ${\boldsymbol{W}}^{\textit{ss}} \in{\boldsymbol{R}}^{d_{u} \times 1024}$, ${\boldsymbol{W}}^{\textit{acc}} \in{\boldsymbol{R}}^{d_{u} \times 1024}$, ${\boldsymbol{W}}^{\textit{fund}} \in{\boldsymbol{R}}^{d_{u} \times 1024}$ and ${\boldsymbol{W}}^{\textit{traf}} \in{\boldsymbol{R}}^{d_{u} \times 1024}$ denote trainable weight matrices for $\textit{seqd}$, $\textit{ss}$, $\textit{acc}$, $\textit{fund}$ and $\textit{traf}$; ${\boldsymbol{b}}^{\textit{emb}} \in{\boldsymbol{R}}^{1024}$ is a trainable bias vector; *relu* is Relu activation function; *batchNorm* represents the batch normalization operation; ${\boldsymbol{W}}^{\textit{pred}} \in{\boldsymbol{R}}^{1024 \times c}$ is the trainable weight matrix used in the classifier network; $c$ is the number of output classes; ${\boldsymbol{b}}^{\textit{pred}} \in{\boldsymbol{R}}^{c}$ indicates the trainable bias vector in the classifier.

The contributions of embeddings in different channels are equally considered at the beginning because their weights are randomly initialized. During the training process, errors between predicted EC numbers and the true labels are evaluated, which are further back propagated to modules in different signal channels, adjusting their weights. Through multiple interactions of training, parameters and contribution weights of different channels are adaptively optimized, improving the final enzyme prediction results.

## EXPERIMENTAL RESULTS

To the best of our knowledge, this is the first attempt to incorporate large language models into deep frameworks as key modules to learn valuable physical properties for accurate enzyme predictions while providing meaningful interpretations. To evaluate the utility of ifDEEPre, we compare it with the state-of-the-art methods in predicting various levels of EC number, e.g. Support Vector Machine (SVM), Logistic Regression (LR), Random Forest (RF), AdaBoost, NN, ResNet, LSTM, EzyPred [16], SVM-Prot [21], ProPythia [18] and ABLE [8]. Accuracy, Precision, Recall, F1-score and Kappa Score are used to evaluate the prediction results. We further analyze protein biological patterns and key motifs learned by ifDEEPre and compare them with the ground truth to examine its implications for downstream applications.

### Automatic detection of enzyme proteins

Enzymes and non-enzyme proteins have apparent differences in catalyzing biochemical reactions. Therefore, level 0 EC number predictions are performed to automatically detect the enzyme proteins from the whole database. We first compare the performances of ifDEEPre with that of the state-of-the-art methods. Experimental results on the NEW and the KNN datasets indicate that ifDEEPre significantly outperforms the state-of-the-art methods, as illustrated in Table 1 and Table 2. ifDEEPre achieves at least 15.55%, 16.36% and 14.22% improvements in Precision, Recall and F1-score for this task on the NEW dataset, compared with the highest scores obtained by the state-of-the-art methods. Meanwhile, on the KNN dataset, ifDEEPre again obtains more accurate results than other methods, e.g. achieving at least 9.79% and 12.33% increases in F1-score and Recall over the state-of-the-art models. Among the baseline models that take sequence encoding (RAW) as inputs, for the NEW dataset, LSTM-RAW achieves the best results in Accuracy and Recall, with scores of 0.861 and 0.853, which however are still, respectively, 15.68% and 16.76% smaller than that of ifDEEPre. We see from Table 1 that for this dataset, ResNet-RAW and AdaBoost-RAW, respectively, achieve the highest F1-score and precision among baseline methods, i.e. 0.854 and 0.824, which are surpassed by ifDEEPre with margins of 16.63% and 20.87%, suggesting strong prediction capacities of ifDEEPre.

**Table 1:** Experimental results in detecting whether a protein is an enzyme on the NEW dataset

Model	Accuracy	Kappa score	Precision	Recall	F1-score
SVM-RAW	0.740	0.759	0.730	0.730	0.830
LR-RAW	0.719	0.738	0.719	0.719	0.719
RF-RAW	0.783	0.792	0.700	0.773	0.793
AdaBoost-RAW	0.802	0.802	0.824	0.853	0.791
NN-RAW	0.849	0.833	0.802	0.830	0.839
ResNet-RAW	0.840	0.893	0.822	0.813	0.854
LSTM-RAW	0.861	0.803	0.819	0.853	0.824
EzyPred	0.913	0.848	0.810	0.801	0.814
SVM-Prot	0.752	0.504	0.754	0.752	0.752
ProPythia	0.901	0.903	0.840	0.856	0.872
ABLE	0.912	0.909	0.862	0.853	0.806
DEEPre	0.965	0.929	0.965	0.965	0.965
ifDEEPre	**0.996**	**0.991**	**0.996**	**0.996**	**0.996**

**Table 2:** Experimental results in detecting whether a protein is an enzyme on the KNN dataset

Model	Accuracy	Kappa score	Precision	Recall	F1-score
SVM-RAW	0.793	0.803	0.773	0.772	0.783
LR-RAW	0.772	0.783	0.753	0.759	0.796
RF-RAW	0.703	0.703	0.784	0.760	0.685
AdaBoost-RAW	0.700	0.681	0.696	0.653	0.702
NN-RAW	0.803	**0.913**	0.813	0.797	0.803
ResNet-RAW	0.800	0.792	0.792	0.762	0.811
LSTM-RAW	0.822	0.767	0.808	0.789	0.813
EzyPred	0.806	0.823	0.820	0.803	0.827
SVM-Prot	0.703	0.406	0.723	0.657	0.689
ProPythia	0.841	0.811	0.849	0.806	0.838
ABLE	0.849	0.801	0.821	0.819	0.834
DEEPre	0.884	0.767	0.879	0.890	0.885
ifDEEPre	**0.920**	0.841	**0.921**	**0.920**	**0.920**

Furthermore, even when compared with the most recent state-of-the-art enzyme prediction methods, e.g. ABLE [8] built upon LSTM neural networks and the attention mechanism, and ProPythia [18] constructed through the combinations of both shallow and deep learning techniques, the proposed ifDEEPre method still achieves more accurate results with large margins on both datasets. For the NEW dataset, among the state-of-the-art methods, ABLE achieves the highest precision, i.e. 0.862, which however is still 15.55% smaller than that of ifDEEPre, i.e. 0.996, as shown in Table 1. For the same dataset, in Recall and F1-score, the prediction results of ProPythia are the best among the state-of-the-art methods with scores of 0.856 and 0.872, which however are, respectively, 16.36% and 14.22% smaller than the scores obtained by ifDEEPre. On the KNN dataset, for the same task, we observe from Table 2 that ABLE and ProPythia, respectively, obtain the highest scores in Recall and F1-score among the state-of-the-art methods with scores of 0.819 and 0.838, which are still much smaller than the scores of 0.920 and 0.920 achieved by ifDEEPre. These results indicate that ifDEEPre can accurately detect enzymes from the whole protein database.

### Annotations on main classes of enzymes

The biocatalytic properties of enzyme proteins vary significantly with their classes. Level 1 EC number prediction experiments are hence conducted to evaluate the capacity of the proposed ifDEEPre method and the state-of-the-art methods in correctly annotating the main classes of the enzyme proteins. The experimental results on the NEW dataset and the KNN dataset are, respectively, demonstrated in Table 3 and Table 4. We observe that the prediction results of ifDEEPre are significantly better than the performances achieved by all the state-of-the-art methods. As shown in Table 3, the Recall and F1-score of ifDEEPre are 0.934 and 0.934, which are, respectively, 13.21% and 8.35% higher than that of ProPythia, and meanwhile are 8.48% and 6.02% higher than that of ABLE, the top two methods in existing enzyme EC number prediction tools.

**Table 3:** Annotation performance comparisons of different predictive tools on the main classes of enzymes on the NEW dataset

Model	Accuracy	Kappa score	Precision	Recall	F1-score
SVM-RAW	0.739	0.718	0.711	0.740	0.725
LR-RAW	0.799	0.752	0.756	0.792	0.773
RF-RAW	0.701	0.713	0.713	0.797	0.703
AdaBoost-RAW	0.794	0.730	0.784	0.791	0.702
NN-RAW	0.843	0.813	0.814	0.826	0.796
ResNet-RAW	0.847	0.813	0.809	0.832	0.802
LSTM-RAW	0.825	0.831	0.822	0.810	0.821
EzyPred	0.853	0.832	0.893	0.846	0.838
SVM-Prot	0.493	0.258	0.504	0.332	0.349
ProPythia	0.895	0.895	0.905	0.825	0.862
ABLE	0.912	0.905	0.916	0.861	0.881
DEEPre	0.912	0.882	0.913	0.882	0.895
ifDEEPre	**0.951**	**0.934**	**0.935**	**0.934**	**0.934**

**Table 4:** Annotation performance comparisons of different predictive tools on the main classes of enzymes on the KNN dataset

Model	Accuracy	Kappa score	Precision	Recall	F1-score
SVM-RAW	0.712	0.753	0.683	0.689	0.712
LR-RAW	0.763	0.763	0.758	0.789	0.772
RF-RAW	0.782	0.771	0.789	0.770	0.782
AdaBoost-RAW	0.801	0.789	0.779	0.782	0.759
NN-RAW	0.821	0.809	0.812	0.801	0.812
ResNet-RAW	0.812	0.813	0.801	0.802	0.802
LSTM-RAW	0.820	0.796	0.781	0.803	0.790
EzyPred	0.838	0.821	0.825	0.817	0.824
SVM-Prot	0.445	0.198	0.506	0.277	0.278
ProPythia	0.835	0.847	0.839	0.847	0.811
ABLE	0.815	0.836	0.803	0.802	0.828
DEEPre	0.903	0.870	0.918	0.862	0.886
ifDEEPre	**0.941**	**0.921**	**0.938**	**0.907**	**0.920**

Meanwhile, we observe from Table 4 that ifDEEPre again significantly outperforms all the rest methods on the KNN dataset, achieving much larger scores in all the evaluation criteria than all the existing methods. For this task on the KNN dataset, the largest accuracy score among all the state-of-the-art methods is 0.838, achieved by EzyPred, which however is 13.48% smaller than that of ifDEEPre, i.e. 0.941. ProPythia obtains the highest precision value of 0.839 among the state-of-the-art methods, which is still much smaller than the score of 0.938 obtained by ifDEEPre, with a giant 11.44% difference. For the same task, the F1-score achieved by ifDEEPre is 0.920, which is 12.80% larger than the highest F1-score of 0.828 among all the state-of-the-art methods, obtained by ABLE. All these results suggest the strong capacity of the proposed ifDEEPre method in correctly annotating the main classes of various enzyme sequences and confirming their functions.

### Annotation on subclasses of various enzymes

**Figure 2:**
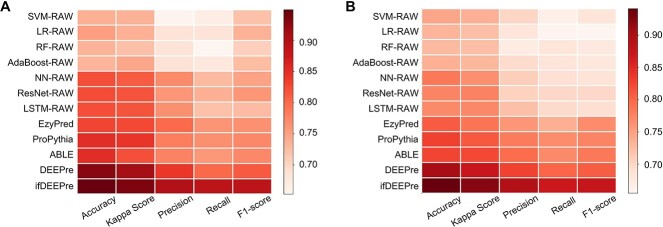
Annotation performance comparisons of ifDEEPre and the state-of-the-art methods on the subclass of various enzymes. (**A**) Annotation results on the NEW dataset. (**B**) Annotation results on the KNN dataset.

Even within a same type of enzyme, different subclasses of enzymes often have diverse functions. Level 2 EC number predictions aim at annotating subclasses of enzymes, given their main classes. The experimental results on the NEW and the KNN datasets are provided in Figure 2. We observe from these figures that ifDEEPre significantly and consistently outperforms the state-of-the-art methods in all the evaluation criteria for both datasets, which indicates the strong prediction capacity of the proposed ifDEEPre method. On the NEW dataset, for the level 2 prediction task, we observe from Figure 2A that the colors of ifDEEPre are much redder than that of others, which indicates that ifDEEPre achieves the highest scores in all the evaluation criteria. The average recall and F1-score values of ifDEEPre achieved for various types of enzyme proteins are 0.890 and 0.890, which are, respectively, 16.19% and 15.14% larger than the highest recall and F1-score values among all the state-of-the-art methods, i.e. 0.766 and 0.773, obtained by ProPythia. This conclusion is also true for the KNN dataset, as demonstrated in Figure 2B. For the KNN dataset, ProPythia obtains the highest accuracy of 0.829, while ABLE achieves the largest Kappa Score and precision of 0.823 and 0.795 among all the comparing models, which however are, respectively, 12.91%, 11.42% and 11.45% smaller than the scores of ifDEEPre, i.e. 0.936, 0.917 and 0.886.

There are several possible reasons. *First*, ifDEEPre can automatically learn rich representations from raw sequences, which provide wide aspects of key information for capturing protein biological properties. Conversely, baseline methods that only consider sequence encoding often obtain poor results. This suggests that extracting rich valuable information from raw sequences is vital for accurate predictions of enzyme functions. *Secondly*, novel self-guided attention is designed to dynamically adjust contributions of representations and fragments according to properties of tasks. Comparing the results of ifDEEPre and ABLE, we find that although both models are constructed upon deep learning, the performances of ifDEEPre are much better. One key difference is that ABLE uses vanilla attention, which learns attention weights from random status and thus may fall into a local optimal solution. Conversely, we design novel self-guided attention to fully consider the diversity in the importance of different elements by dynamically detecting key elements and adjusting their roles, hence learning more accurate weights to promote performances. *Lastly*, by introducing a large protein language model trained through hundreds of millions of proteins into our deep framework, ifDEEPre can effectively incorporate valuable expertise on biological properties of proteins, e.g. contact patterns, function information and mutational effects. Such important information spans the evolutionary diversity of tremendous protein data and thus effectively extends biological knowledge of the entire neural network, further improving the final enzyme prediction performances.

### Influences of learned representations

**Figure 3:**
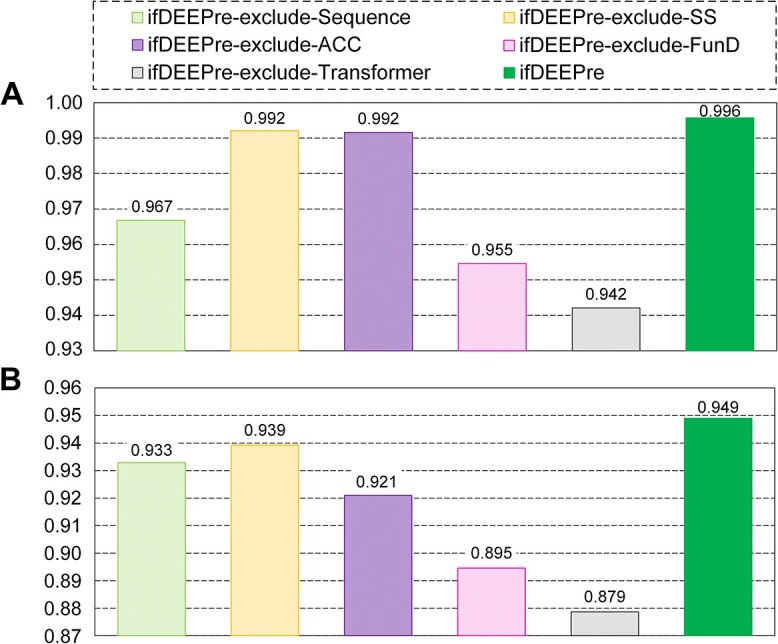
Influences of each automatically learned representation on various enzyme function prediction tasks of the NEW dataset. (**A**) Enzyme protein detection results. (**B**) Annotation results of enzyme subclass.

To explore the influences of learned features on enzyme predictions, we conduct ablation studies on the variants of ifDEEPre by sequentially excluding each representation. The ablation study results on the NEW dataset are shown in Figure 3, evaluated via accuracy. Some interesting phenomena are observed. Significant effectiveness of transformer features is discovered, whose exclusion makes results drop apparently. When transformer features are removed, the magnitudes in accuracy declines caused by this factor are the most apparent among all the ablation studies. The exclusion of the transformer features makes the accuracy in detecting enzymes decrease from 0.996 to 0.942 on the NEW dataset, as shown in Figure 3A. Meanwhile, we see from Figure 3B that this operation makes accuracy drop from 0.949 to 0.879 in the task of enzyme subclass annotations. These results indicate the importance of transformer features for accurate enzyme function predictions. This is because transformer features are extracted via a large language module, which is trained via hundreds of millions of proteins and covers different aspects of biological information. Therefore, the incorporation of such valuable expertise effectively extends biological knowledge of the whole neural network, capturing vital protein biological knowledge spanning evolutionary diversities, e.g. secondary structure, contact patterns, function information and mutational effects, which significantly enhances the generalization ability of ifDEEPre for new protein sequences.

Functional domain is vital for the precise predictions of enzyme functions, the exclusion of which causes significant decreases in the performances. For the NEW dataset, when the functional domain is excluded, the accuracy for annotating subclasses of enzymes drops from 0.949 to 0.895, as shown in Figure 3B. These experimental results indicate the vital roles of functional domain plays in enzyme predictions. This is because such representation provides key information for specifying the start and the end positions for each domain, which is quite useful for the accurate analysis of proteins. Sequence encoding has moderate influences on enzyme function predictions, the exclusion of which leads to a certain drop in the prediction performances. For the NEW dataset, we observe from Figure 3A that when sequence encoding is excluded, the accuracy in detecting enzymes decreases from 0.996 to 0.967. These results indicate that sequence encoding, which contains sequential patterns of proteins, reflects certain key information for improving the enzyme prediction results.

Minor influence of secondary structure is observed, which reflects structure information of proteins and its exclusion causes certain decreases in enzyme predictions. For the NEW dataset, excluding secondary structure causes slight drops in prediction accuracy, e.g. decrease from 0.949 to 0.939 for the task of annotating enzyme subclasses, as shown in Figure 3B. These results suggest that local folding information contained in the secondary structure representations of proteins can provide certain properties of protein functions. However, secondary structure information might have been already included in the transformer features learned from millions of proteins, so its exclusion has minor influences. Solvent accessibility also has limited influences. When solvent accessibility is not considered, the accuracy for the NEW dataset drops from 0.949 to 0.921 on the task of enzyme subclass annotation, as shown in Figure 3B. We see that the influences of solvent accessibility are also minor, which is probably because transformer features extracted from massive proteins reflect complete knowledge of protein properties, including information on the openness of a local region of proteins, thus weakening the impacts of solvent accessibility.

Please note that the main target of this study is to build an accurate and fast framework to provide convenient enzyme prediction services to the public. The extraction of the PSSM features through PSI-BLAST takes a relatively long time for each sequence [9], around 14 s, which would be huge when the number of proteins is large. Conversely, the use of HMMER to acquire functional domains and RaptorX Toolbox to obtain secondary structure features is much faster, respectively, around 0.47 and 2.22 s for each sequence. Therefore, when constructing this deep framework, we exclude PSI-BLAST while keeping HMMER and RaptorX. In addition, the choice of the comparative database in HMMER searches may influence the model’s performance. Thus, to exactly evaluate the influences of the protein language module, we utilize the same HMMER searching database as that used in our previous work DEEPre [9]. Meanwhile, to provide fast and convenient enzyme prediction servers to the public, we further exclude RaptorX Toolbox when building the web server of ifDEEPre, significantly decreasing the average scanning time per sequence with very minor drops in performance.

### Impacts of designed structure and attention

**Figure 4:**
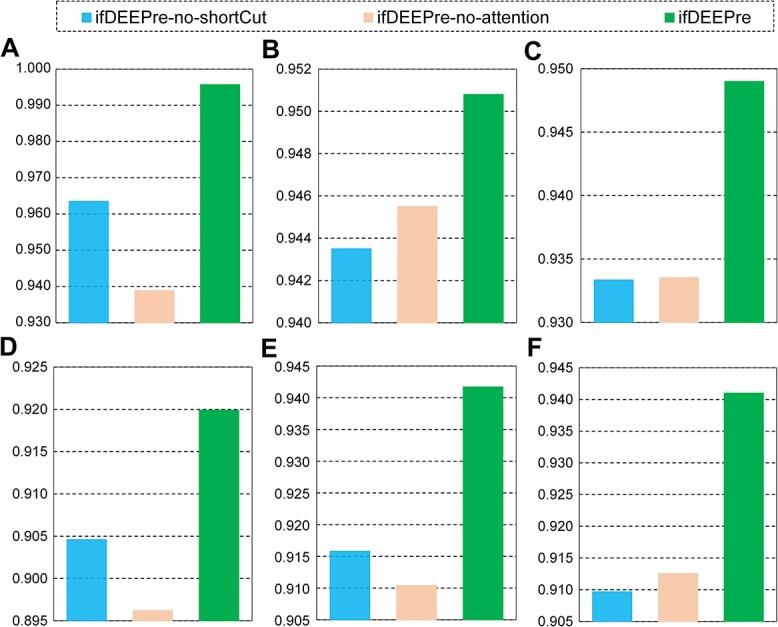
Importance of the designed network structure and attentive learning for various enzyme prediction tasks. (**A–C**) Level 0, 1 and 2 results on NEW dataset. (**D–F**) Level 0, 1 and 2 results on the KNN dataset.

To explore the effectiveness of the designed channel-wise short-cut connection structure and self-guided attention for EC number predictions, we compare the performances of ifDEEPre with its two variants, ifDEEPre-no-shortCut and ifDEEPre-no-attention, which take the same representations as inputs with ifDEEPre. Therefore, the differences between the results of ifDEEPre and its two variants are caused by the designed attention mechanism and short-cut learning structures. The designed short-cut learning structure is effective for improving enzyme function predictions, the removal of which causes large drops in performances for both the NEW and the KNN datasets, as shown in Figure 4A, B, C, D, E, and F. For the task of detecting enzymes in the NEW dataset, removing this structure leads to a decrease in the accuracy from 0.996 to 0.963, which illustrates the importance of the designed channel-wise short-cut connection. For annotating the main classes of enzymes in the KNN dataset, when the short-cut structure is excluded from the whole network, the accuracy experiences a large decline from 0.941 to 0.916, as shown in Figure 4E. These results indicate the removal of the channel-wise short-cut structures from the network causes significant decreases in performances even when inputs and the rest modules remain the same, proving the importance of this structure for accurate enzyme predictions. This is because signal channels in the designed framework are constructed via deep learning techniques that contain a large number of neural layers to increase learning capacities, which however may potentially impair information propagation along long channels and severely impact final results. By introducing the residual learning structure into each signal channel, short-cut connections provide highways for the smooth passage of crucial information, effectively facilitating information propagation and stabilizing the training of the deep network to improve final results.

The incorporation of the designed self-guided attentive learning mechanism meanwhile significantly promotes various enzyme function prediction performances. The removal of this mechanism causes giant decreases in the results for both datasets. For the level 0 task of the NEW dataset, the accuracy drops from 0.996 to 0.939 after removing self-guided attention from the ifDEEPre neural network, as illustrated in Figure 4A. This attention mechanism has significant influences on the KNN dataset as well. This removal operation on the KNN dataset leads to an apparent decrease in the accuracy for the task of annotating the main classes of various enzymes, dropping from 0.941 to 0.910, as demonstrated in Figure 4E. These experimental results indicate that the proposed self-guided attention mechanism plays an important role in achieving accurate analysis for enzyme functions, the removal of which could cause significant decreases in the prediction performances. The reason is that this mechanism can effectively learn accurate and personalized attention weights by fully considering the biological properties of different sequence representations. Thus, it can dynamically adjust the contributions of different representations, optimizing their influences according to the unique characteristic of the prediction task to improve final results. Furthermore, this mechanism demonstrates the contribution weights of all the amino acid fragments, indicating their unique influence scores and improving the understanding on protein data to promote real-world applications.

### Cross-dataset test on third-party dataset

**Figure 5:**
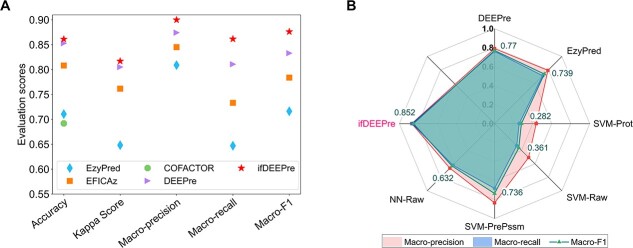
The performance comparisons of different servers on various levels of EC number prediction tasks of the third-party independent and non-overlapping dataset, i.e. the COFACTOR dataset. ifDEEPre again significantly outperforms all the existing servers under the cross-dataset setting. (**A**) Level 1 of EC number prediction results on the COFACTOR dataset. (**B**) Level 2 of EC number prediction results on the COFACTOR dataset.

To evaluate the generalization ability of the proposed method, we compare the performances of various servers in predicting the level 1 and level 2 EC numbers of a third-party independent and non-overlapping dataset. We conduct such cross-dataset experiments on the COFACTOR benchmark dataset, which is considered to be a difficult dataset in the field of enzyme function predictions [20]. To ensure the test data have enough diversity with the training data and there is no bias in the results, we eliminate the sequences in the COFACTOR dataset that overlap with the training data of ifDEEPre (the NEW dataset). Please note that the ifDEEPre model trained through the NEW dataset is used as the final version to construct the web server and perform predictions for the COFACTOR dataset because the KNN dataset does not contain the third digit EC number and cannot be used to train the sub-sub-class models to predict the full four EC number digits. Since it takes about 4 h to run COFACTOR for one sequence to get the prediction results, we directly report the original experimental results from their paper. The first-digit and the second-digit EC number prediction results are provided in Figure 5, which suggest the significant superiority of ifDEEPre over the existing enzyme function prediction tools. For the level 1 task, ifDEEPre achieves a 16.92% increase over COFACTOR in accuracy, as shown in Figure 5A. Considering that COFACTOR requires the 3D structures of enzymes, while ifDEEPre only needs sequence information, this improvement is significant. Meanwhile, ifDEEPre achieves at least 9.22% and 12.83% improvements in F1-score and Recall compared with the best results among all these enzyme function prediction servers.

Meanwhile, ifDEEPre significantly outperforms all the existing methods in the level 2 EC number predictions of the COFACTOR dataset, as illustrated in Figure 5B. The F1-score of ifDEEPre for this task is 0.852, which is, respectively, 10.64%, 15.28%, 15.72% and 34.77% larger than the scores of 0.770, 0.739, 0.736 and 0.632, obtained by the top four models among the state-of-the-art methods, i.e. DEEPre, EzyPred, SVM-PrePssm and NN-Raw. At the same time, DEEPre, EzyPred, SVM-PrePssm and NN-Raw, respectively, achieve the four highest recall scores among the state-of-the-art methods, i.e. 0.761, 0.713, 0.682 and 0.619, which however are still 13.52%, 21.04%, 26.60% and 39.46% smaller than the recall score of 0.864, achieved by ifDEEPre. These results indicate that ifDEEPre keeps its powerful enzyme function prediction capacities even for unseen sequences in a third-party independent and non-overlapping dataset, suggesting its strong robustness and generalization ability. This is probably because ifDEEPre incorporates a large language module trained via hundreds of millions of proteins into the designed framework as a key component. Such modules introduce vital protein biological knowledge on contact patterns, structure knowledge and function information spanning evolutionary and species diversities. As a result, even when tested on new and previously unseen protein sequences, ifDEEPre still achieves much more accurate EC number prediction results than all the state-of-the-art methods.

### Robustness of ifDEEPre against similarity

The limitation on the redundancy between the test data and the training data is vital for evaluating the utility of the designed enzyme prediction framework. Therefore, we perform similarity analysis on the COFACTOR dataset to evaluate the robustness of ifDEEPre toward different degrees of sequence similarities. There are two popular tools for reducing sequence redundancy between two protein datasets, i.e. MMseqs2 [22] and CD-HIT [23]. Because the lowest similarity allowed by CD-HIT for reducing sequence redundancy between protein datasets is 40% [23], this tool cannot be used to remove all the sequences that exceed the similarity 30%. Therefore, we use MMseqs2 [22] to remove the redundant sequences in the COFACTOR dataset by successively reducing the similarity threshold from 80% to 30% with the interval of 10%, through the similarity comparisons with the training data (the NEW dataset).

**Figure 6:**
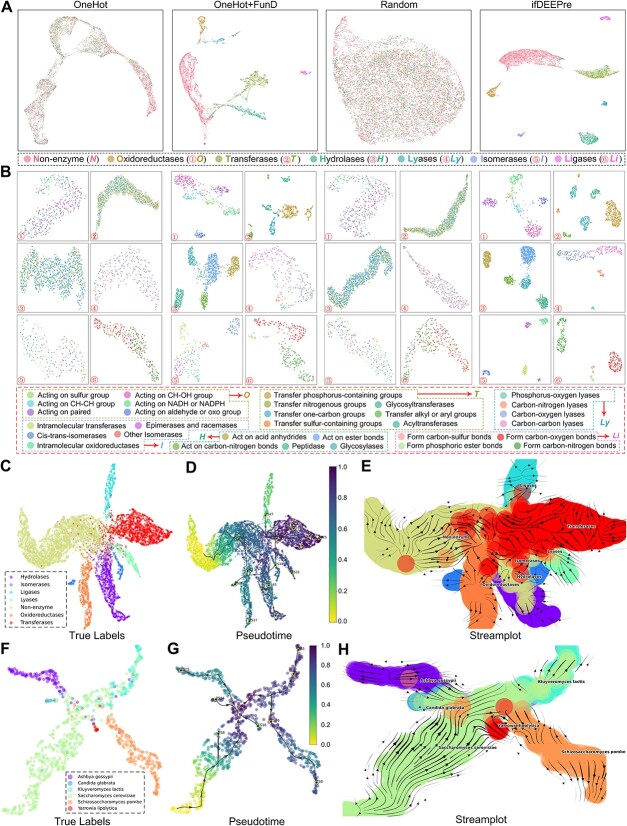
Number of proteins in the COFACTOR dataset under different sequence similarity thresholds with the training data.

The number of the proteins left in the COFACTOR dataset under different similarity thresholds with the training data is shown in Figure 6. We see that as the similarity threshold decreases gradually, proteins left in the COFACTOR dataset become fewer, because the conditions used to reduce sequence redundancy become increasingly strict. For the very small similarity threshold 30%, because this data processing condition is very strict, there are only three proteins left. Therefore, although ifDEEPre achieves very accurate results for enzyme predictions under this similarity, e.g. obtaining scores of 1.0, 1.0, 1.0, 1.0 and 1.0 in Accuracy, Kappa Score, Precision, Recall and F1-score for annotating the main classes of enzymes, which are much larger than other methods, e.g. 0.667, 0.400, 0.333, 0.333 and 0.333 obtained by DEEPre, this result is not included to avoid the biased results caused by such few protein samples.

**Figure 7:**
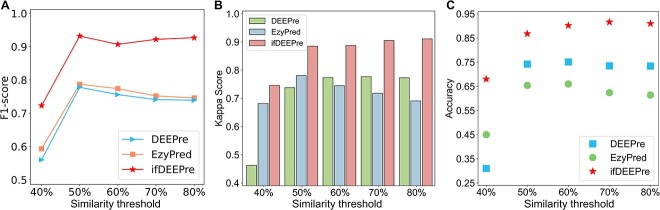
The performances of the proposed ifDEEPre method and the state-of-the-art methods in annotating the main class and the sub-class of enzymes in the COFACTOR dataset under different sequence similarity thresholds with the NEW dataset, which suggests that ifDEEPre outperforms the existing methods under various similarities. (**A**) The changes of F1-scores of different methods along with sequence similarities in annotating the main class of enzymes. (**B**) The Kappa score of the enzyme main class annotations achieved by different methods under various sequence similarities. (**C**) The changes in the Accuracy scores of different methods along with sequence similarities in annotating the sub-class of enzymes.

The comparisons of the enzyme prediction results obtained by ifDEEPre and the state-of-the-art methods under similarity thresholds from 40% to 80% are shown in Figure 7, which suggest the superiority of ifDEEPre over the existing methods. Please note that although the training data of the state-of-the-art method EzyPred [16] are not the NEW dataset, to exactly compare the prediction performances of ifDEEPre and EzyPred, we still use the final version of EzyPred to perform enzyme predictions for the same left sequences under each similarity threshold. Therefore, the prediction results of EzyPred may not drop with the decreases in the similarity threshold of the COFACTOR dataset with the NEW dataset. Figure 7A demonstrates the changes of the F1-scores of ifDEEPre, DEEPre and EzyPred with the similarity thresholds for annotating the main class of enzymes. We see that the F1-score of ifDEEPre is consistently and significantly larger than that of other models under various similarities, which suggests the strong prediction power and robustness of ifDEEPre. The comparisons of the Kappa score among these models in annotating the main class of enzymes under various similarity thresholds again suggest the superiority of ifDEEPre, as shown in Figure 7B. The annotation results of ifDEEPre, DEEPre and EzyPred for the sub-class of enzyme proteins in the COFACTOR dataset under various levels of similarity thresholds are provided in Figure 7C. We observe that the enzyme prediction results of ifDEEPre are again consistently better than those of other models under different similarities in predicting enzyme sub-classes. These results demonstrate the strong prediction capacities and robustness of ifDEEPre under various similarity settings.

### ifDEEPre captures multi-level protein biological knowledge

To demystify what biological information has been learned by the proposed ifDEEPre method and the physical meanings of model outputs, we take a further look at the protein biological knowledge contained in the ifDEEPre embedding.

**Figure 8:**
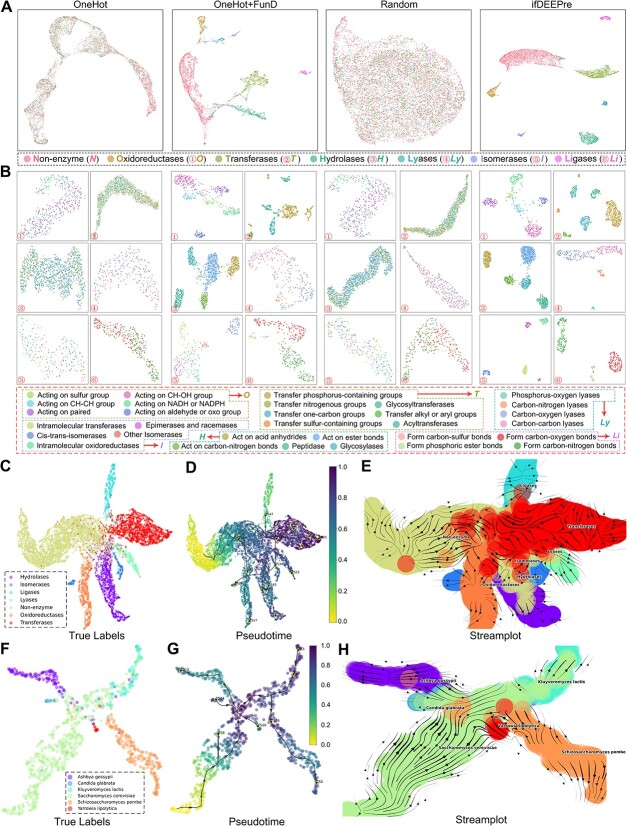
ifDEEPre encodes multi-level protein biological patterns and successfully infers the evolutionary directions of enzymes and the evolutionary trajectories of yeast sub-specie. (**A**) Enzyme Atlas with all the enzyme families obtained via different embedding ways. (**B**) Detailed distributions of each enzyme family (*i.e.*, Oxidoreductase, Transferase, Hydrolase, Lyase, Isomerase, and Ligase) all suggest the effectiveness of the designed ifDEEPre framework in shaping the representations of various enzyme families. (**C-E**) ifDEEPre provides rich knowledge in inferring the evolutionary directions of enzymes, which positively contributes to the design of new effective enzyme products. (**F-H**) ifDEEPre accurately predicts the evolutionary trajectories of yeast sub-species, which are highly consistent with the ground-truth discovered by expensive and time-consuming lab-based biological experiments.

**Functions of enzymes** vary along with the biological types, typically determined by the physical properties. ifDEEPre is expected to capture such vital biological properties in the embeddings generated from protein sequences. We construct the enzyme Atlas using the embeddings of different levels to evaluate the effectiveness of ifDEEPre in capturing multi-level protein patterns. UMAP [24] is used to project the embeddings to 2D plane by reducing their dimensions. First, to explore the *effectiveness of the proposed framework in shaping the representations*, we compare the visualization results of ifDEEPre with the one-hot encoding model (OneHot) as well as the one-hot encoding and functional domain model (OneHot+FunD), as shown in Figure 8A. We see that the projections of OneHot are only roughly grouped, while elements of different enzyme types are severely mixed, which indicates that the OneHot model can barely learn accurate protein information. Although the results of OneHot+FunD improve to some extent, its embeddings still have no clear boundaries while some groups still have the issue of jumbled elements, e.g. non-enzyme group and Hydrolases enzyme. In contrast, the results of ifDEEPre are well grouped for different enzymes with clear boundaries, which suggests that embeddings generated by ifDEEPre well distinguish the biological properties of different enzymes and thus can make precise inferences on their functions. This is probably because ifDEEPre seamlessly learns valuable protein biological properties from hundreds of millions of protein data by introducing a large language model as a key module. The incorporation of such vital biological information effectively extends the physical knowledge of the whole neural network, e.g. secondary structures, contact patterns and function information, significantly promoting the inference of unique characteristics of each enzyme group and the learning of meaningful embeddings.

Secondly, we explore the *influences of the learning process in shaping the representations*. The visualization results of the embeddings generated by ifDEEPre and the randomly initialized ifDEEPre (Random) are provided in Figure 8A. We observe that the projections of embeddings for different types of enzymes generated by the Random model are mixed, which indicates that the representations of the Random model can hardly capture useful enzyme biological knowledge. As a consequence, the Random model fails to distinguish the physical properties of different enzyme sequences. In contrast, after the optimization process, the embeddings of ifDEEPre can be easily grouped with very clear boundaries, which proves the effectiveness of the designed end-to-end learning mechanism adopted in the proposed deep prediction framework. This is because in the training process, all the parameters in different components of ifDEEPre are jointly optimized in an end-to-end way, which allows parameters to be timely adjusted according to the biological properties in the tasks until reaching a very small negligible prediction error. Therefore, the embeddings generated by ifDEEPre can capture the biological properties of different protein groups and accordingly build very precise enzyme Atlas based on the learned useful physical knowledge.

**Atlas of enzyme sub-groups** are meanwhile explored. To take a closer look at the performances of ifDEEPre in learning biological knowledge on the unique properties of different sub-groups under each enzyme type, we further visualize projections results of embeddings generated by ifDEEPre, OneHot, OneHot+FunD and Random for all the sub-groups within each enzyme. Learning useful representations for enzyme sub-groups is much harder because the number of samples in each enzyme sub-group is smaller than that in the main group, which may cause difficulties in the optimization of model parameters. The visualization results of all the sub-groups under each enzyme group are provided in Figure 8B. We observe that the projections of ifDEEPre embeddings are much clearer than those of other models, indicating that ifDEEPre successfully learns accurate representations that reflect the unique biological properties of enzymes in each sub-group. Even for Phosphorus-oxygen lyases under the Lyase enzyme group, which have very small data size and are difficult to learn accurate representation from such few samples, ifDEEPre still achieves projection that shows clear boundaries with other sub-groups (Figure 8B4). In contrast, the projection results of Lyase obtained by other models have either severely the mixed-samples issues (e.g. OneHot and Random) or very unclear boundaries (e.g. OneHot+FunD). These results suggest the successful learning of useful biological knowledge in the embeddings of the designed ifDEEPre framework. Such key biological information is learned from hundreds of millions of proteins, which effectively improves the capacity of ifDEEPre to capture the high-level structure and function properties of various enzyme groups and sub-groups. Thus, even for enzyme sub-groups with few samples, ifDEEPre still learns precise biological knowledge, successfully establishing accurate Atlas for different enzyme sub-groups.

**Enzyme evolutionary trajectories** are meanwhile studied. We utilize a popular single-cell transcriptomics trajectory inference technique, VIA [25], to capture the evolutionary directions of enzymes by taking ifDEEPre embeddings as inputs. To provide a complete and accurate evolutionary map of the trajectories of enzymes, we perform such analysis on the whole enzyme dataset, which can be grouped into different categories by considering the unique biological properties of each type of enzyme. First, ifDEEPre embeddings are generated for different types of enzyme sequences. Then, VIA is utilized to infer the evolutionary trajectory of enzymes based on ifDEEPre embeddings (Figures 8C–E). We see from stream-plot that ifDEEPre can distinguish enzyme proteins of different families (Figure 8C), which is consistent with the conclusions of the strong prediction ability of ifDEEPre, drawn from above enzyme Atlas analysis.

Furthermore, the embeddings generated by ifDEEPre can present the evolutionary relationships among different enzyme families. One interesting phenomenon is observed between Transferases and Lyases, i.e. parts of evolutionary directions of Transferases are the same with that of Lyases (bottom right of Figure 8E). Considering that several Transferases enzymes, e.g. Nu-class glutathione S-transferase, can act as Lyase enzymes under certain conditions, e.g. glutathione lyase, they may share some similar properties in catalytic chemical reactions [26], suggesting correctness of the learned evolutionary map. Meanwhile, we observe from this map that the distance between the Hydrolase family and the Isomerase family is relatively small, as shown in the bottom middle of Figure 8E. The possible reason is that Hydrolases and Isomerases contain subunits with similar sizes and they can act upon substrates with similar structures [27]. Lastly, we see that the distances between non-enzyme proteins and enzyme proteins are giant, which suggests that ifDEEPre precisely distinguishes the biological properties of enzymes from that of non-enzymes in catalyzing chemical reactions. These results indicate that ifDEEPre captures the biological patterns of non-enzyme and enzyme proteins, hence correctly grouping them into correct families. Furthermore, the embeddings generated by ifDEEPre reflect the unique physical properties of various enzyme families, which provide rich information in inferring the evolutionary directions of enzymes and can positively contribute to the design of novel effective enzymes.

**ifDEEPre facilitates yeast evolutionary study.** As an important microbial species, yeast provides many enzymes, vital for medical and industrial applications. Detailed studies on the chemical properties of enzymes from different yeasts and their evolutionary trends are crucial for the wider usage of yeasts. Hence, we investigate the extended application of utilizing ifDEEPre to predict the evolutionary directions of different yeast sub-species. The trained ifDEEPre model is first used to generate embeddings for different yeast sub-species. Since our ifDEEPre framework incorporates valuable knowledge on the physical properties of proteins learned from hundreds of millions of sequences, we assume that ifDEEPre embeddings extracted from protein sequences of various yeast sub-species can reflect their unique biological characteristics, effectively promoting the studies of yeast sub-species evolution.

Evolutionary trends of yeast sub-species predicted by ifDEEPre are shown in Figures 8F–H, highly consistent with the ground-truth. We see that the evolutionary trends between two very ancient yeast sub-species, i.e. Saccharomyces cerevisiae and Schizosaccharomyces pombe, are relatively independent because they are located at two distant positions on the map with different evolutionary directions. This is consistent with existing findings that although Saccharomyces cerevisiae and Schizosaccharomyces pombe diverged from a common ancestor about 1000 million years ago, they evolved toward two different directions [28, 29]. As another distant clade in the yeast evolutionary map, the last common ancestor of Yarrowia lipolytica existed with Saccharomyces cerevisiae over 300 million years ago [30]. As a result, there are many differences between the properties and functions of Yarrowia lipolytica and Saccharomyces cerevisiae, e.g. the antisense gene widely exists in Saccharomyces cerevisiae and its progenies, but such genes are not found in Yarrowia lipolytica [30]. As illustrated in Figure 8H, the large genetic differences between these two yeast sub-species are successfully captured by the yeast evolutionary map inferred by our method.

In contrast, the evolutionary relationships of Saccharomyces cerevisiae and the rest three yeast sub-species, i.e. Candida glabrata, Kluyveromyces lactis and Ashbya gossypii, are much closer, as illustrated in Figure 8H. This is probably because these sub-species originated from a common ancestor shared by Saccharomyces cerevisiae at a more recent time than Schizosaccharomyces pombe and Saccharomyces cerevisiae, e.g. the common ancestor of Ashbya gossypii and Saccharomyces cerevisiae existed about 100 million years ago, 90% more recent than that of Schizosaccharomyces pombe [31]. This suggests that sub-species that started to evolve toward different directions at more recent time tend to have more common genomes and closer evolutionary relationships. We observe that the trajectory relationships between Saccharomyces cerevisiae and Candida glabrata are also close. Although they have totally different real-world application values and influences, e.g. Saccharomyces cerevisiae is often used for winemaking and baking while Candida glabrata causes diseases, e.g. Candida infections, their close phylogenetic relationships are proved [32, 33]. Such discovery is useful because it identifies potential drug resistance properties shared by these two sub-species, assisting doctors in designing effective treatments to heal Candida infections [34]. These results indicate that ifDEEPre embeddings contain rich valuable evolutionary information on different sub-types of yeasts, which improves the accurate inferences of yeast sub-species evolutionary trends and potentially promotes the discovery of novel yeast subspecies.

### ifDEEPre detects key amino acid motifs of a peroxidase in the NEW dataset

**Figure 9:**
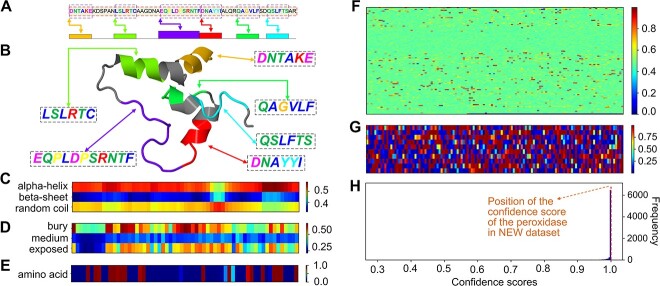
ifDEEPre detects key protein motifs of a peroxidase enzyme in NEW dataset and provides meaningful explanations on the contributions of different fragments and structures, which are highly consistent with the knowledge discovered by biomedical experts. (**A**) Detected key motifs and fragments from the whole peroxidase sequence. (**B**) Motif analysis in 3D protein structure. (**C**) Attention weights learned for secondary structure. (**D**) Attention weights learned for solvent accessibility. (**E**) Attention weights learned for sequence encoding. (**F**) Attention weights learned for functional domains. (**G**) Attention weights learned for transformer embedding. (**H**) Location of the analyzed peroxidase in the whole confidence distribution of enzyme prediction results on the NEW dataset.

To evaluate the advantages of using ifDEEPre for protein analysis, we visualize attention weights learned for all the representations, which demonstrate the contributions of all the elements, improving understanding on the results to increase interpretations. The interpretability analysis for a peroxidase sequence in the NEW dataset is shown in Figure 9. The amino acid sequence of the studied enzyme is ‘DNTAKEKDSPANLSLRTCAAGDNAEQPLDPSRNTFDNAYYIALQRQAGFSDQV LSLFTSAR’, which is a peroxidase with the EC number 1.11.1.7 (Figure 9A). This enzyme catalyzes redox reactions, playing key roles in biomedical and industrial applications, e.g. energy production, drug delivery devices and bioremediation [35]. Peroxidase enzymes are mainly collected from plant tissue, e.g. the cells of vitis rotundifolia. The 3D structure of the peroxidase enzyme generated by AlphaFold [36] is provided in Figure 9B, which significantly catalyzes reactions that break up peroxides via a free radical mechanism. In biological processes, peroxidases donate electrons to ascorbic acid and ferricyanides, hence transforming them into harmless products [37]. Our ifDEEPre method correctly predicts the main class of this protein enzyme with a confidence score of 0.99999833 by only taking the amino acid sequence as input. The location of this score in the confidence score distributions of the whole prediction results is given in Figure 9H, which belongs to the most frequent range of all the scores.

Attention weights learned for secondary structure indicate that alpha-helix is vital for accurate analysis of peroxidase sequences and predictions of their functions (Figure 9C), which is consistent with existing studies [38]. This suggests that when analyzing the structures and functions of protein, more attention is suggested to be paid to alpha-helix information, which helps obtain more precise results. Figure 9D provides the attention weights learned for solvent accessibility of the peroxidase, which shows that the top two largest scores are given to the bury and the exposed states. This is reasonable because both states have been proven to be key factors in describing the openness of local regions of proteins, which significantly impact their functions [39]. This indicates that specific descriptions on the openness of a local region about a protein (buried or exposed) often convey more valuable information on the structure and functions of proteins than a vague one (medium). The attention weights learned for sequence encoding of the protein are shown in Figure 9E, which indicates that different amino acid fragments often possess diverse degrees of importance in determining the catalytic activity of the peroxidase. This is reasonable because many studies have proved that the modifications of certain regions in peroxidase proteins can significantly improve the catalytic efficiency while the modifications of other fragments have very minor influence [40, 41].

Furthermore, to evaluate whether ifDEEPre can detect key amino acid fragments in protein sequences, we utilize MEME [42] to identify motifs and compare the locations of these motifs with that of important regions detected by our model. The motifs identified by MEME are given in Figure 9A and the corresponding 3D structure is shown in Figure 9B. First, by comparing Figures 9A and 9C, we see that locations of key regions identified for the most important secondary structure information, i.e. alpha-helix, match well with that of motifs, e.g. the most vital region learned for alpha-helix is on the right side, which perfectly matches the position of motif ’QSLFTS’. Meanwhile, vital regions learned for random coil and beta-sheet are located at center right, which corresponds to the position of motif ’DNAYYI’ well. Secondly, important regions identified for solvent accessibility match well with the positions of key motifs, e.g. the top two vital regions of the bury state, center and center left are the same with locations of motifs ‘EQPLDPSRNTF’ and ‘LSLRTC’ (Figures 9A and 9D). Thirdly, important fragments identified for sequence encoding, which locate on the center left and center right, also match with that of motifs ’LSLRTC’, ’QAGVLF’ and ’QSLFTS’, as indicated in Figures 9A and 9E. Furthermore, compared with the relatively simple motif analysis tools, ifDEEPre provides richer information on the roles of every element. Specifically, by clearly illustrating the contributions of all the fragments, ifDEEPre provides vital information for selecting suitable candidates of segments for editing operations, significantly improving the efficiency in designing more effective enzymes.

We then visualize attention weights learned for the functional domain, which is a 16306D vector. For the convenience of visualization, zeros are appended to this vector until it reaches the length of 16 384, which is reshaped to a $128 \times 128$ matrix, as shown in Figure 9F. We see that different regions of the weights learned for functional domain have diverse degrees of importance. Therefore, to achieve accurate enzyme function predictions, it is important to focus attention on important regions. Attention weights learned for transformer features are provided in Figure 9G, which indicates that many learned weights are in color RED, suggesting that the majority of transformer features are crucial for the enzyme function prediction tasks. This is consistent with the previous analysis that transformer features contain rich valuable protein biological knowledge, which can well distinguish the unique physical characteristics of various enzyme groups and improve the function prediction results.

### ifDEEPre detects key amino acid motifs of an exonuclease in the KNN dataset

**Figure 10:**
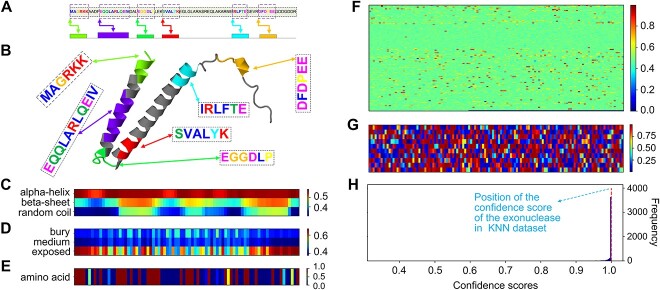
ifDEEPre detects key motifs of an exonuclease in the KNN dataset and provides meaningful explanations on the contributions of different fragments and structures, which are highly consistent with the knowledge discovered by biomedical experts. (**A**) Detected key motifs and fragments from the whole exonuclease sequence. (**B**) Motif analysis in the 3D protein structure. (**C**) Attention weights learned for secondary structure. (**D**) Attention weights learned for solvent accessibility. (**E**) Attention weights learned for sequence encoding. (**F**) Attention weights learned for functional domains. (**G**) Attention weights learned for transformer embedding. (**H**) Location of the studied exonuclease in the whole confidence distribution of the enzyme prediction results on the KNN dataset.

The amino acid sequence of the studied protein in the KNN dataset is ‘MAGRKKAADFEQQLARLQEIVDALEGGDLPLEKSVALYKEGLGLARASREQLAKARNEIRLFTEGEVRDFDP EEGDDGDDR’, which is an exonuclease enzyme with EC number 3.1.11.6 (Figure 10A). This type of enzyme is mainly collected from the cells of desulfovibrio vulgaris subsp. The 3D structure of this enzyme obtained via AlphaFold is given in Figure 10B, which can degrade single-stranded DNA into several large acid-insoluble oligonucleotides and further degrade them into small oligonucleotides [43]. ifDEEPre correctly predicts the main class of this exonuclease protein by only taking its raw sequence as input, achieving a large confidence score of 0.9999974, which is located in the most frequent score range of the distributions of confidences (Figure 10H).

The attention weights learned for the secondary structure features of the exonuclease are provided in Figure 10C, which again indicates that alpha-helix is important for the analysis of proteins. As shown in Figure 10D, the attention weights learned for solvent accessibility of the exonuclease demonstrate that the exposed state is more concerned, which is consistent with existing discoveries [44]. The attention weights learned for the encoding features of the exonuclease show that ifDEEPre distinguishes the diversity in the importance of different amino acid fragments (Figure 10E). Meanwhile, we see that there are some differences between important fragments of exonuclease and peroxidase, which indicates that different enzymes may play their unique functions via diverse segments. Vital motifs identified by MEME are given in Figures 10A and B, which are highly consistent with the locations of the important fragments automatically detected by ifDEEPre. The most important regions identified by ifDEEPre for alpha-helix of secondary structure are located close to that of key motifs ‘EQQLARLQEIV’, ’EGGDLP’ and ’IRLFTE’. Meanwhile, the positions of the detected vital fragments in the exposed state of solvent accessibility are very close to that of the motifs ‘MAGRKK’ and ‘DFDPEE’. Figure 10F shows weights learned for functional domain, which also indicates that different regions have diverse importance, suggesting that more attention should be paid to regions assigned with large weights to achieve accurate analysis. Attention weights learned for transformer features of the exonuclease also indicate the importance of this type of representation for the function prediction of exonuclease (Figure 10G). These results again suggest the effectiveness of ifDEEPre in detecting key motifs from long sequences, further improving the interpretability of the results.

### Superiority of ifDEEPre over DEEPre

We further build a practical website version of ifDEEPre only using transformer features and functional domain, termed **ifDEEPre-web**, and develop it into an easy-to-use web server to provide convenient enzyme prediction services to the public, available at https://proj.cse.cuhk.edu.hk/aihlab/ifdeepre/. This web server provides detailed descriptions about the architecture of ifDEEPre, sample input and sample outputs. Meanwhile, definitions of different enzyme main classes and sub-classes are provided to map each EC number with the biological functions. In addition, prediction results of all the proteins in the input file are displayed one by one with gray lines as separations, where external links are provided for describing the functions of the predicted EC number, making the results easy to understand. Last but not least, the enzyme prediction results of our webserver can be conveniently downloaded for future studies of users.

**Figure 11:**
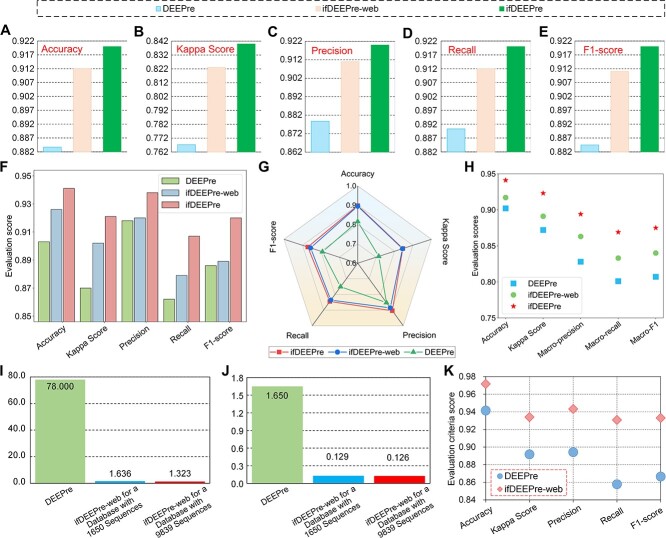
Superiority of ifDEEPre and ifDEEPre-web over DEEPre. (**A–E**) Performance comparisons of DEEPre, ifDEEPre-web and ifDEEPre on level 0 task of KNN dataset. (**F**) Comparisons of DEEPre, ifDEEPre-web and ifDEEPre on level 1 task of KNN dataset. (**G**) Comparisons of DEEPre, ifDEEPre-web and ifDEEPre on joint level 0 and 1 task of KNN dataset. (**H**) Comparisons of DEEPre, ifDEEPre-web and ifDEEPre on level 2 task of KNN dataset. (**I**) Comparisons of time needed for scanning protein database between ifDEEPre-web and DEEPre, where the unit of time is second/per sequence (s/per s). (**J**) Comparisons storage space needed for scanning protein database between ifDEEPre-web and DEEPre, where the unit for storage space is million bytes/per sequence (MB/per s). (**K**) Performance comparisons of DEEPre and ifDEEPre-web on level 3 task of NEW dataset.

The comparisons between ifDEEPre, ifDEEPre-web and the original version DEEPre [9] in various levels of EC number predictions, running speed and storage space requirement are demonstrated in Figure 11. The prediction performances of ifDEEPre and ifDEEPre-web on **various EC number predictions** are significantly better than the results of DEEPre (Figures 11A–H). In the task of detecting enzymes in the KNN dataset, Kappa scores of ifDEEPre and ifDEEPre-web are 0.841 and 0.823, which are, respectively, 9.65% and 7.30% larger than the score of 0.767 obtained by DEEPre (Figure 11B). Meanwhile, in the task of annotating main classes of enzymes for this dataset, the Kappa score of DEEPre is 0.870, which is also smaller than that of ifDEEPre and ifDEEPre-web, i.e. 0.921 and 0.902 (Figure 11F). We observe from Figure 11G that ifDEEPre and ifDEEPre-web obtain the F1-score of 0.871 and 0.856 for the joint level 0 and 1 task, which, respectively, outperform DEEPre with 10.06% and 8.16% improvements. For the task of annotating subclasses of enzymes in this dataset, ifDEEPre and ifDEEPre-web also, respectively, achieve 7.97% and 7.61% higher precision scores than DEEPre (Figure 11H). These results suggest the effectiveness of the designed novel network structure and learning mechanism in improving the prediction capacity for enzyme functions.

The comparisons between **running speed** of ifDEEPre-web and DEEPre in scanning proteins are given in Figure 11I, which indicates that the time needed by ifDEEPre-web for each sequence is about 58.96 times smaller than that of DEEPre. This is because DEEPre is designed to perform this task in serial ways, which repeats the operations of loading large trained models to extract features and perform enzyme predictions for every single sequence, which is slow when handling a large number of proteins. Meanwhile, DEEPre relies on PSSM features, which take long time to extract. Average time of DEEPre for scanning each protein is around 78 s, which rapidly increases with the number of query sequences. Conversely, ifDEEPre-web scans databases in fast parallel ways, which learns features of sequences with equal length in batches, saving much time spent in repeatedly loading trained models when scanning large protein databases. The prediction stages of ifDEEPre-web are meanwhile designed in parallel ways to jointly perform function predictions for sequences in each class, which again saves much time. Therefore, with the increases in the data sizes of protein databases, the average time needed by ifDEEPre-web for scanning per sequence will further decrease because of the increases in the number of sequences in each batch. This can be verified from Figure 11I, i.e. average scanning time of ifDEEPre-web for a database with 1650 proteins is 1.636 s and will further decrease to 1.323 s for a database with 9839 proteins, respectively, 47.68 and 58.96 times smaller than that of DEEPre. These results indicate the significant advantages of ifDEEPre-web in scanning large protein databases.

Furthermore, ifDEEPre-web needs a much smaller **storage space requirement** than DEEPre. For each protein, DEEPre needs a storage space of around 1.65 million bytes (MB) to store the extracted features, while that required by ifDEEPre-web is only about 0.126 MB, 13.10 times smaller (Figure 11J). This is vital for the function predictions of large-scale protein databases, e.g. for 10 million proteins, the storage space required by DEEPre is 16.5 T, which however is only 1.269 T for ifDEEPre-web. Based on the above analysis, we can conclude that the proposed method significantly outperforms the original version DEEPre in various aspects, including prediction accuracy, speed and storage space requirement. In short, this method achieves more accurate results than DEEPre and is 58.96 times faster while only needing 7.64% of the storage space of DEEPre, which significantly increases the capacity to analyze large protein databases.

To evaluate the utility of ifDEEPre-web in enzyme function predictions, we further utilize it to perform **level 3 and level 4 EC number predictions**, providing the full four digits of the EC system to specify complete functions of proteins. We first analyze the performances of ifDEEPre-web in the level 3 EC number predictions, which aim at annotating the sub-subclass of the input enzymes when given the main class and subclass. The results of ifDEEPre-web for this task of the NEW dataset are shown in Figure 11K, which indicates that ifDEEPre-web achieves scores of 0.972, 0.934, 0.943, 0.931 and 0.933 in accuracy, Kappa score, precision, recall and F1-score. These results indicate that the proposed method can also achieve accurate predictions for the third digit of EC numbers, e.g. the F1-score and Recall of ifDEEPre-web are, respectively, 7.68% and 8.51% larger than that of DEEPre. Regarding the level 4 EC annotation task, i.e. predicting the sub-sub-subclass of input sequences, more data are needed to train models. This is because the number of samples in each sub-sub-subclass of the dataset is too few, not enough to optimize the parameters of models and may cause unreliable results. Therefore, we utilize phmmer [45] to search for the fourth digit from the database, thus obtaining the full four EC numbers by combining it with the previously predicted three digits.

## DISCUSSION AND CONCLUSIONS

In this study, we present ifDEEPre, a new deep learning-powered framework for the interpretable, accurate and fast predictions of EC numbers. Extensive experimental results on various levels of enzyme function predictions on multiple datasets demonstrate that ifDEEPre outperforms the state-of-the-art methods by a large margin in all the evaluation metrics. In the task of detecting the enzymes from all the proteins in the NEW dataset, ifDEEPre achieves more than 15.55%, 16.36% and 14.22% improvements in Precision, Recall and F1-score, compared with the best prediction results among all the state-of-the-art methods. Meanwhile, when annotating the main classes of proteins in the KNN dataset, the accuracy of ifDEEPre is 0.941, which is 12.29% larger than the score of 0.838 obtained by EzyPred, the highest accuracy among all the state-of-the-art methods. For the joint level 0 and 1 task, and the annotation task of the subclasses of enzymes, ifDEEPre also significantly outperforms the state-of-the-art methods, e.g. achieving at least 12.82% improvements in Kappa score on the KNN dataset and 18.06% increases in F1-score on the class 2 enzyme of the NEW dataset, compared with the best results in the existing models. These experimental results indicate that ifDEEPre can consistently and significantly outperform the state-of-the-art methods in various enzyme function prediction tasks. The proposed ifDEEPre method has some significant advantages over the existing models.

To the best of our knowledge, this is the first attempt to introduce a large language model into deep enzyme function prediction frameworks as a key computational module to learn valuable physical properties of proteins from hundreds of millions of sequences, effectively extending biological knowledge of the whole neural network while avoiding increasing computational burden. This design effectively incorporates rich types of biological knowledge spanning evolutionary diversity into ifDEEPre, including secondary structure, contact patterns, function information, mutational effects and folding information, which boost the final EC number prediction results. The incorporation of this large protein language module significantly improves the prediction accuracy from 0.942 to 0.996 in the task of detecting enzymes from the KNN dataset and from 0.879 to 0.952 in the level 2 EC number prediction task of the NEW dataset. More importantly, this module effectively promotes the evolutionary inference performances of many enzymes and species. Meanwhile, we demonstrate the effectiveness of ifDEEPre in predicting the evolutionary relationships between various yeast sub-species, which are highly consistent with the ground truth discovered by expensive and time-consuming lab-based biological experiments.

To effectively optimize the contributions of different representations and motifs according to unique characteristics of prediction tasks, we design a novel self-guided attention mechanism to dynamically adjust the attention weights of all the elements in the whole training stage. This mechanism can automatically reinforce the roles of key elements and detect important motifs from protein sequences to provide meaningful interpretations, assisting biological scientists in designing more effective strategies. Experimental results demonstrate that the designed self-guided attention mechanism significantly increases the accuracy in the enzyme detection task on the NEW dataset from 0.939 to 0.996 and meanwhile improves the joint level 0 and 1 EC number prediction accuracy on the KNN dataset from 0.865 to 0.898. Furthermore, interpretability analysis studies on a peroxidase from the NEW dataset and an exonuclease from the KNN dataset both indicate that the proposed method can successfully detect key amino acid motifs and fragments from the whole long protein sequence in a learnable way, which are highly consistent with existing discoveries while providing some novel discoveries for researchers to further explore. These analyses illustrate that ifDEEPre can clearly demonstrate the roles of all the amino acid elements, which provides valuable information for choosing suitable segments for editing operations, improving the efficiency in designing novel enzyme products.

Furthermore, to meet the needs of modeling large number of proteins in the post-genomic era, we carefully screen representations learned from raw protein sequences to ensure the running speed of the entire framework, thus excluding all the manually crafted features and the PSSM features, which take a long time to extract. In addition, we design ifDEEPre-web for scanning databases in fast parallel ways, which learns features of sequences with equal length in batches and performs function predictions for sequences of one class simultaneously. As a result, the average time of using ifDEEPre-web to scan a database with 1650 sequences is only 1.636 s and such time further decreases to 1.323 for a database with 9839 sequences, which are, respectively, 47.68 and 58.96 times smaller than the time needed by the DEEPre model. This design meanwhile significantly decreases the storage space requirement of ifDEEPre-web. To be specific, for each protein sequence, DEEPre needs a storage space of around 1.65 million bytes (MB) to store the extracted features, while the space required by ifDEEPre-web is only about 0.126 MB, which is 13.10 times smaller. In short, this method achieves much better results than DEEPre, but only needs about 1.70% of the running time and 7.64% of the storage space, which significantly increases its capacity in analyzing large protein databases and mining valuable biological knowledge to promote its applications.

We believe that the proposed ifDEEPre method and its faster version ifDEEPre-web can serve as powerful tools for the accurate, interpretable and fast analysis of large-scale proteins, which positively contributes to the tasks of detecting enzymes, annotating their class, capturing protein knowledge and identifying key amino acid motifs. This framework can promote the discovery of novel and more effective enzymes to improve public health and industrial production. There are four possible future research directions. First, we will expand ifDEEPre by integrating more protein knowledge, e.g. 3D structures of proteins [46]. This would further improve the prediction results and promote the biological learned by this framework to promote downstream tasks. Secondly, we intend to introduce the small-sample learning theory [47] to perform accurate and robust predictions of the fourth digit of the EC number in a data-driven and learnable way. Thirdly, the relationships between the amino acid motifs of enzymes detected in this framework and their enzymatic reactions will be explored. This research direction would meanwhile promote the development of protein designs [48]. Lastly, we will further optimize the HMMER and the RaptorX components. We may further design new modules, e.g. Graph Neural Networks, to replace HMMER and RaptorX to extract functional domain and protein structure knowledge in machine learning ways, developing even faster and more accurate enzyme prediction tools.

**Key Points**

- We propose a novel tool named ifDEEPre that can achieve more accurate enzyme function predictions than existing methods while automatically detecting key motifs to provide meaningful interpretations.
- We strictly screen representations learned from raw sequences to improve the running speed of the whole deep framework, 50 times faster than DEEPre while requiring 12.89 times smaller storage space.
- Extensive experimental results and analysis are presented to illustrate the superiority and the Interpretability of the proposed ifDEEPre method for enzyme function predictions.
- We illustrate that trained ifDEEPre models can capture multi-level protein biological patterns and accurately infer the evolutionary trends of enzymes by only taking raw sequences without label information.
- Meanwhile, ifDEEPre accurately predicts the evolutionary relationships between different yeast sub-species, which are highly consistent with the ground-truth discovered by expensive and time-consuming biological experiments.

## Author Biographies

**Qingxiong Tan** is a postdoctoral fellow at the Department of Computer Science and Engineering at The Chinese University of Hong Kong. His research interests include health care, protein and RNA structure predictions.

**Jin Xiao** is a PhD student at the Department of Computer Science at Hong Kong Baptist University. Her research interests include bioinformatics.

**Jiayang Chen** is a research asistant at the Department of Computer Science and Engineering at The Chinese University of Hong Kong. His research interests include protein and RNA structure predictions.

**Yixuan Wang** is a PhD student at the Department of Computer Science and Engineering at The Chinese University of Hong Kong. Her research interests include protein structure prediction and bioinformatics.

**Zeliang Zhang** is a PhD student at the Department of Computer Science at University of Rochester. His research interests include bioinformatics.

**Tiancheng Zhao** is a Master student at the School of Software at Shandong University. His research interests include bioinformatics.

**Yu Li** is an asistant professor at the Department of Computer Science and Engineering at The Chinese University of Hong Kong. His research interests include healthcare and bioinformatics.
